# Leukocyte count in COVID-19: an important consideration

**DOI:** 10.1186/s43168-020-00045-8

**Published:** 2020-11-18

**Authors:** Samah Selim

**Affiliations:** Departrment of Chest Diseases, Faculty of Medicine, Cairo University, Cairo, Egypt

To the Editor,

Dear Sir,

In December 2019, an outbreak of pneumonia associated with high mortality was noticed in Wuhan city, China. Thereafter, the World Health Organization (WHO) confirmed the novel coronavirus named SARS-CoV-2 was responsible for the clinical features and declared this as COVID-19 [1].

The virus attaches to angiotensin-converting enzyme-2 (ACE) receptors on various types of cells including type 2 pneumocytes, renal epithelial cells, and lymphocytes. Furthermore, the virus can lead to exaggerated inflammatory response known as cytokine storm that is characterized by increased interleukin (IL)-2, IL-7, granulocyte colony-stimulating factor (GCSF), interferon-γ inducible protein 10, monocyte chemo-attractant protein-1, macrophage inflammatory protein 1-α, and tumor necrosis factor-α. It is responsible for the development of acute respiratory distress syndrome (ARDS) and multiple organ failure [2–4].

Several studies demonstrated that neutrophilia that might be related to the cytokine storm (absolute neutrophil count above the normal range; 3–7.5 × 10^9^/L) and/or lymphocytopenia (lymphocyte count < 1.5 × 10^9^/L) were present in severe cases of COVID-19 pneumonia and were associated with poor prognosis. Neutrophil to lymphocyte ratio (NLR) has also been found to predict disease severity in the early stages of SARS CoV-2 infection. In contrast, small studies reported a significant reduction in granulocytes in severe as compared to non-severe patients [5].

In conclusion, from the previous evidence, the following points should be taken in consideration:

1. Patients with COVID-19 pneumonia may have normal (4–11 × 10^9^/L), low, or high leukocyte count. This may further help in following the progression of the disease and help in the decision regarding treatment strategy. Physicians must be aware of the cytokine storm and avoid the use of granulocyte colony-stimulating factor for the leukopenia associated with SARS CoV-2 as it may worsen the condition with early development of ARDS.
2. Lymphocytopenia is a reliable indicator of early SARS CoV-2 infection and helps in tracing of contacts besides assessment of disease progression along the course of COVID-19 pneumonia. Further studies are needed to evaluate its role in immune-compromised patients especially those having human immune-deficiency syndrome. Declaration of the effect of SARS CoV-2 on different subsets of T lymphocytes warrants future studies.
3. The interpretation of NLR in the follow-up of patients with COVID-19 should consider the use of corticosteroids and the occurrence of bacterial co-infection that could interfere with the result.

## Data Availability

Not applicable

## Abbreviations

WHO: World Health Organization
SARS-CoV-2: Severe acute respiratory syndrome coronavirus-2
COVID-19: Coronavirus disease 2019
ACE: Angiotensin-converting enzyme
IL: Interleukin
GCSF: Granulocyte colony-stimulating factor
ARDS: Acute respiratory distress syndrome
NLR: Neutrophil to lymphocyte ratio
